# Predictors of Trust and Engagement in Personalized Healthcare: A Study of AI-Driven Diagnosis and Treatment in Saudi Arabia

**DOI:** 10.3390/healthcare14131954

**Published:** 2026-07-02

**Authors:** Howeida Abusalih, Amaal Alqahtani, Kady Alsarhan, Layan Alshehri, Khafoq Aldosari, Ymna Alqahtani, Shatha Abohimed

**Affiliations:** Department of Health Sciences, College of Health and Rehabilitation Sciences, Princess Nourah Bint Abdulrahman University, Riyadh 11671, Saudi Arabia

**Keywords:** personalized healthcare, diagnosis, treatment, artificial intelligence, Large Language Models (LLMs), Saudi Arabia, user trust, patient decision-making, AI reliance

## Abstract

**Background:** Driven by Vision 2030, Saudi Arabia is rapidly integrating Artificial Intelligence into its healthcare ecosystem. This study investigates the patterns, predictors, and sociodemographic determinants of AI reliance and dependence in healthcare decision making, focusing on how trust influences the shift toward personalized digital diagnosis. **Methods:** A cross-sectional study was conducted with 627 adults in Saudi Arabia using convenience sampling. Data collected via online questionnaires were analyzed using JMP student edition version 18 software to evaluate user interaction with symptom checkers, wearables, and generative AI. A multidimensional framework assessed how trust and dependence influence health-seeking behaviors. **Results:** The findings reveal high AI engagement, with 63.7% of respondents using AI tools weekly. Conversational AI and LLMs are the dominant interfaces (92.2%), primarily serving as “gatekeepers” for personalized diagnosis (71.6%) and treatment suggestions (76.9%) before formal consultations. While gender significantly impacts reliance (*p* = 0.0037), trust was identified as the only significant predictor of overall engagement (*p* < 0.0001). Notably, age, education, and income had no statistical impact (*p* > 0.05), indicating uniform adoption across groups. **Conclusions:** For surveyed cohorts, trust is the primary determinant of AI reliance, overriding traditional demographic factors. Fostering user trust is essential for the successful implementation of AI-driven personalized healthcare solutions.

## 1. Introduction

Artificial Intelligence (AI) has emerged as a transformative driver in modern science and engineering, encompassing a diverse spectrum of subfields ranging from general cognitive processes, such as learning and perception, to highly specialized applications, including disease detection and the verification of complex mathematical theorems. As a useful discipline, AI is increasingly being integrated into nearly every facet of intellectual life, acting as both a hub of knowledge and an active agent in problem-solving [1]. The utility of AI in healthcare decision-making became remarkably prominent during the COVID-19 pandemic, where it played a vital role in disease monitoring and diagnostic screening, thereby demonstrating its profound value in enhancing clinical and public health outcomes [2].

In the modern era, the search for medical advice has moved beyond the “Dr. Google” era of static search results into the era of “conversational health-seeking.” Despite this accessibility, the transition toward AI-mediated health seeking is accompanied by significant concerns regarding clinical accuracy and patient safety. Empirical evaluations have indicated that global digital assistants, such as Siri, Alexa, and Google Assistant, may provide incomplete or potentially misleading responses to critical health inquiries, often failing to recognize the distinction required for acute medical emergencies [3]. Recent evidence from Alharbi et al. (2025) suggests that while advanced models like ChatGPT exhibit encouraging performances, demonstrating alignment with clinical guidelines in specialized areas such as ophthalmic chemical injuries, rigorous clinical oversight remains critical to ensure diagnostic reliability [4].

Modern AI applications for the general public have transitioned from simple digital archives to active decision-support systems, which are broadly categorized into three domains: symptom checkers and triaging, wearable biometrics, and generative AI. Applications such as Ada, WebMD, and K Health utilize probabilistic graphical models to compare user-input symptoms against vast medical databases, often performing at accuracy rates comparable to general practitioners in non-emergency scenarios. Simultaneously, wearable devices like the Apple Watch, Fitbit, and Oura ring have shifted from passive step-counting to active “preventative” monitoring, utilizing AI to detect arrhythmias (e.g., atrial fibrillation) or sleep apnea. Finally, the emergence of LLMs such as ChatGPT and Gemini has introduced a layer of synthesized, personalized advice. In the context of the general population, the use of AI for health-related decision making is defined as the autonomous utilization of technologies, specifically LLMs, symptom checkers, and wearable biometrics, to interpret symptoms and determine health-seeking behaviors without immediate professional intervention [5].

Reliance on AI in health-related decisions refers to the psychological and behavioral commitment to act upon AI-generated results. This transition from familiarity to dependence is heavily influenced by a spectrum of factors ranging from supplementary support to delegated decision-making. While many users utilize AI for initial triaging or convenience (e.g., appointment reminders), a notable group maintains consistent use for chronic disease self-management. Reliance is not dictated by accuracy alone; it is shaped by task complexity and cognitive load. Users may follow AI advice despite limited understanding of system limitations if the interface provides a high degree of perceived “humanness” or empathy [6]. However, reliance is often condition-specific, with higher dependence observed in routine self-management compared to life-saving health decisions [7]. Populations in Saudi Arabia are more likely to act on AI outputs when the system is framed as clinically validated or embedded within trusted institutional portals, such as national e-health platforms [8].

Trust serves as a multidimensional psychological state that bridges the gap between AI output and user action. In the Saudi context, it is categorized into three dimensions: Competence Trust, Integrity Trust, and Benevolence Trust. Competence Trust refers to the belief in the AI’s ability to provide accurate clinical advice [9]; Integrity Trust involves the expectation that the system operates honestly and discloses limitations—explainability is a critical factor here, with users prioritizing transparency over raw performance; and Benevolence Trust is the perception that the system acts in the user’s best interest, often mediated by data security and privacy concerns [10].

In KSA, 61.1% of users believe AI assists professionals, yet only 12.5% believe it can replace them, indicating that Competence Trust is conditional [11].

Patients now bypass formal settings to use AI in evaluating health risks and selecting treatment options independently, as shown by several studies [12]. Nevertheless, even when AI aligns with clinical guidelines, the absence of human oversight can lead to inappropriate self-treatment and delays in seeking professional care.

Regionally, systematic reviews across Arab nations demonstrate a robust integration of Artificial Intelligence (AI) into clinical healthcare routines. In Saudi Arabia specifically, the exigencies of the COVID-19 pandemic served as a critical catalyst, accelerating adoption rates to an estimated 82% [13]. Despite the widespread assumption that “digital natives” are more receptive to new tech, demographic patterns reveal a surprising divergence; while younger generations possess higher general technical literacy, research by Cinalioglu et al. identified a paradoxical trend: older adults reported significantly greater comfort in utilizing AI for health-related decision making compared to their younger counterparts [14].

In spite of this high penetration, public engagement in Saudi Arabia is characterized by a “trust–competence paradox”, as shown by a study conducted by Alshutayli, who found that 52.3% of respondents express comfort in using “AI doctors” as partial alternatives to human physicians, where 63.7% remain concerned about their ability to accurately communicate symptoms to an algorithm [15]. Furthermore, while a significant portion of the population believes AI will enhance professional performance, a study conducted by Syed et al. showed that only 12.5% believe it can fully replace a physician [16].

### 1.1. Public Health Significance

This study addresses the urgent need to map the circumstances under which the Saudi public utilizes AI, distinguishing between helpful integration and hazardous substitution of professional expertise. By identifying the drivers of this trust–reliance gap, this research provides the evidence base necessary to align Vision 2030’s technological ambitions with robust patient safety protocols and optimized resource allocation.

### 1.2. Aim

This study aims to investigate the determinants of AI reliance in healthcare decision-making. By analyzing user trust and demographic variables, the research seeks to understand how AI facilitates personalized diagnosis and treatment planning among individuals in Saudi Arabia.

## 2. Materials and Methods

### 2.1. Study Design, Setting, and Period

A quantitative, descriptive cross-sectional study with inferential analysis was conducted in the Kingdom of Saudi Arabia (KSA), and the data were collected between January and March 2026. The formal study and full implementation strictly adhered to the protocol following IRB approval, leading to the finalization of the report in May 2026. This design was chosen to provide a representative snapshot of the prevalence, perceptions, and levels of reliance on Artificial Intelligence (AI) in health-related decision making during the nation’s current phase of rapid digital transformation. KSA serves as an ideal setting due to its diverse population of approximately 35 million and its strategic focus on AI under Vision 2030 [17].

### 2.2. Participants and Eligibility Criteria

The target population comprised adults (≥18 years) residing across all five administrative regions of KSA (Northern, Southern, Eastern, Western, and Central). Inclusion Criteria: Residents of KSA (citizens and expatriates), regular users of internet-enabled digital devices, and those providing electronic informed consent. Exclusion Criteria: Individuals with cognitive or psychiatric impairments, healthcare professionals and medical students (to eliminate professional knowledge bias), and participants involved in the pilot phase.

### 2.3. Sampling and Sample Size

#### 2.3.1. Sampling

Non-probability convenience sampling was utilized, and the survey was hosted on Google Forms and disseminated via major social media platforms (WhatsApp, X, and LinkedIn) to ensure a broad geographical reach across the Kingdom.

#### 2.3.2. Sample Size Determination

The minimum sample size was calculated using the single population proportion formula [18].

$$n=\frac{Z^{2}P\left(1-P\right)}{d^{2}}$$

where 52.3% of respondents reported comfort in using AI as a physician alternative, agreeing with a previous study [8]. With a 95% confidence level (*Z* = 1.96) and a 5% margin of error (*d* = 0.05), the initial requirement was 384 participants. To account for the design effect (DEFF) of web-based non-probability sampling, a factor of 1.5 was applied (384 times 1.5 = 576). The target was rounded to 580, and a final sample of 627 was obtained (representing an 8% buffer for non-response).

### 2.4. Instrumentation and Adaptation

#### 2.4.1. Data Collection Instrument

A structured, self-administered Arabic questionnaire was developed, comprising four sections:

Sociodemographic: age, gender, education, income, occupation, region, and marital status.

AI Usage Patterns: frequency of use, tool types (e.g., ChatGPT, chatbots), and motivations (e.g., cost-saving, convenience).

Perceptions and Self-Efficacy: adapted from Zhang et al. [19], assessing the trustworthiness of AI advice.

Reliance and Dependence: AI reliance was measured using the framework by Cao and Huang [20], while emotional/behavioral dependence was assessed via the AI dependence scale by Morales-García et al. [21].

Scoring: items used a 5-point Likert scale (1: Strongly Disagree to 5: Strongly Agree). Reliance and Dependence: Total scores (range 4–20) were categorized into low (4–9.33), moderate (9.34–14.66), and high (14.67–20). Treatment Trust: Total scores (range 4–8) were categorized into low (4–5.33), moderate (5.34–6.66), and high (6.67–8).

#### 2.4.2. Instrument Translation and Cultural Adaptation

To ensure linguistic and conceptual equivalence, the original English scales were translated into Arabic following a rigorous forward-and-backward translation protocol. First, two independent bilingual researchers native to Saudi Arabia translated the questionnaire into Arabic (forward translation), and then a third independent bilingual researcher translated this Arabic draft back into English (back-translation) without having access to the original English version. The back-translated version was compared against the original English text by the research team to resolve any semantic discrepancies.

Cultural adaptation was subsequently performed by the expert panel in healthcare informatics and public health to ensure the phrasing aligned with local terminology and cultural contexts in Saudi Arabia. Finally, the tool was pilot-tested on a small sample (*n* = 23) to verify clarity and readability before formal data collection commenced. Survey on the Use of AI-Based Health Tools and Health Decision-Making Among Adults was presented in Supplementary Materials.

A multi-step validation process was employed to ensure the instrument’s psychometric integrity. Because the measurement items were adapted directly from established, previously validated frameworks, specifically Zhang et al. [19] for trustworthiness, Cao and Huang [20] for AI reliance, and Morales-García et al. [21] for AI dependence, construct validity had been rigorously established by the original authors. Therefore, a de novo Exploratory Factor Analysis (EFA) or Confirmatory Factor Analysis (CFA) was not performed. To adapt these scales to the target population, a forward-and-backward translation process into Arabic was conducted by independent bilingual researchers. Content and face validity were then evaluated by a panel of experts in healthcare informatics and public health to ensure cultural relevance, clarity, and alignment of the items with the underlying constructs. A pilot study (*n* = 23) was conducted to confirm linguistic clarity; pilot data were excluded from the final analysis. Internal consistency for the full sample (*n* = 627) was excellent: the total 8-item scale demonstrated a Cronbach’s alpha of 0.907, while the AI reliance and AI dependence subscales yielded alpha values of 0.830 and 0.880, respectively.

### 2.5. Data Analysis

Data was analyzed using JMP software (SAS JMP, Cary, NC, USA). Descriptive statistics (frequencies, percentages, means, and standard deviations) summarized the demographic profile and AI usage. Inferential analysis included chi-square tests for categorical variables, and ANOVA and independent *t*-tests for comparing mean scores. A multivariable linear regression model identified independent predictors of AI reliance, and statistical significance was set at *p* < 0.05.

A multiple linear regression analysis was conducted to identify the primary predictors of AI reliance. The dependent variable was operationalized as the total continuous score from the AI reliance subscale, while the independent variables entered into the model simultaneously included baseline demographic factors (age, gender, education, and income) alongside continuous scores from the Treatment Trust Scale.

### 2.6. Ethical Considerations

This study was conducted in accordance with the Declaration of Helsinki, and ethical approval was obtained from Princess Nourah bint Abdulrahman University (25-0689). Participants provided informed consent electronically before accessing the survey.

### 2.7. Declaration of Generative AI Use

Generative AI was used in this study solely for the purpose of refining the manuscript’s language, grammar, and formatting to ensure clarity and professional standards. It was not used for study design, data collection, or the interpretation of statistical results.

## 3. Results

### 3.1. Sociodemographic Characteristics of Participants

The study sample (*n* = 627) was predominantly female (64.7%) and notably skewed toward a younger demographic, with 67.8% of respondents aged 18–24. Consistent with this age profile, the majority were students (66%) and single (77.3%). Academically, 55% of participants hold a diploma or bachelor’s degree. While all administrative regions of Saudi Arabia were represented, the Central Region accounted for the largest proportion (41.3%). The sample consisted almost entirely of Saudi nationals (97.1%), with a diverse distribution across monthly income brackets.

### 3.2. AI Tool Utilization and Engagement Patterns

Table 1 shows that the baseline adoption patterns of Artificial Intelligence (AI) tools for health-related inquiries among the sample (*n* = 627) are comprehensive of diverse behavioral frequencies and software choices. A substantial portion of the cohort engages with AI tools dynamically: 26.3% (*n* = 165) utilize them daily and 37.2% (*n* = 233) access them several times a week. Conversely, only 7.6% (*n* = 48) reported rarely or never using AI for health-related inquiries. Large Language Model (LLM) chatbots (e.g., ChatGPT, Gemini) emerged as the overwhelmingly dominant tool category, utilized by 92.2% (*n* = 579) of the sample. Health and fitness applications were used by 22.8% (*n* = 143), while specialized symptom checkers (3.0%, *n* = 19) and general health recommendation systems (4.6%, *n* = 29) were less frequently adopted. In terms of clinical sequencing, 71.6% (*n* = 449) of respondents use these tools directly for self-diagnosis purposes. Similarly, 71.8% (*n* = 450) deploy AI applications as a preliminary screening step prior to scheduling formal medical consultations. Financial motivations are more divided; 46.7% (*n* = 293) utilize AI explicitly as a cost-avoidance measure to bypass professional medical charges, whereas the majority (53.3%, *n* = 334) do not see financial avoidance as a driving factor.

### 3.3. Determinants of AI Adoption and Behavioral Engagement

The specific motivations driving users to integrate AI tools into their personalized healthcare journeys are outlined in Table 2. The prominent drivers for utilization were seeking initial treatment suggestions (76.9%, *n* = 482), obtaining lifestyle and nutrition recommendations (73.0%, *n* = 458), using tools for preliminary consultation before visiting a doctor (71.8%, *n* = 450), and executing independent self-diagnosis (71.6%, *n* = 449). Additionally, comparing medications and understanding clinical risks motivated 69.2% (*n* = 434) of respondents. Financial parameters were less uniform; 46.7% (*n* = 293) utilized AI explicitly to avoid professional medical visit costs, while 53.3% (*n* = 334) rejected cost-avoidance as a core catalyst for them. Synthesizing these metrics into structural engagement archetypes (Table 3) reveals high general interaction. The overall frequency index for health-related AI decision making achieved a high mean score of 3.75 out of 5. This active interface is further demonstrated by the 71.6% agreement rate for self-diagnosis and the 71.8% rate for clinical pre-screening behaviors.

### 3.4. Distribution of User Trust, Reliance, and Dependence

Evaluating psychometric constructs across the sample reveals notable distinctions in how users perceive and rely on health AI technology (Table 4). Both AI reliance (mean = 15.025, SD = 3.24) and AI dependence (mean = 14.695, SD = 3.63) exhibited high average scores relative to their maximum possible scores of 20. Conversely, the General Treatment Trust was established at a more moderate tier (mean = 6.489, SD = 0.92, max. score = 8). Categorical stratification based on predefined score thresholds (Table 5) shows that the majority of users experience high levels of engagement. Specifically, 59.8% (*n* = 375) of the population exhibited high AI reliance, and 55.7% (*n* = 349) demonstrated high psychological or behavioral AI dependence. Moderate engagement was observed in roughly one-third of the population (34.4% for reliance, 35.4% for dependence), while low-tier engagement remained rare across both dimensions (5.8% and 8.9%, respectively).

### 3.5. Sociodemographic Determinants of AI Reliance

Bivariate analysis using Pearson’s chi-square tests was performed to examine the relationships between key sociodemographic traits and structural AI reliance tiers (Table 6). Gender emerged as a statistically significant factor (chi-square test = 11.196, *p* = 0.0037), showing that females represented a higher absolute volume of the high-reliance category (*n* = 249) compared to males (*n* = 126). Conversely, age groups showed no statistically significant differences in reliance behaviors (chi-square = 9.066, *p* = 0.5258), though the 18–24 age category contained the largest concentration of high-reliance individuals (*n* = 264). Similarly, formal education levels did not display a significant relationship with reliance choices (chi 2 = 2.694, *p* = 0.6103).

### 3.6. Correlation and Regression Analyses

#### 3.6.1. Correlation Matrix

To analyze the relationships between the underlying continuous psychometric variables, a Pearson linear correlation matrix was calculated (Table 7). A strong, positive, and statistically significant correlation was observed between AI reliance and AI dependence (r = 0.7308, *p* < 0.01). Treatment Trust also displayed positive correlations with both AI reliance (r = 0.3843, *p* < 0.01) and AI dependence (r = 0.4476, *p* < 0.01).

#### 3.6.2. Regression Diagnostics

Prior to modeling, standard linear regression assumptions were verified. Multicollinearity assessments yielded high tolerance metrics (all values greater than 0.20) and low Variance Inflation Factors (VIFs ranging safely between 1.15 and 2.40), well below the conservative threshold of 5.0, confirming the data were free from multicollinearity. Residual analysis via visual inspection of normal P-P plots and homoscedasticity scatterplots confirmed normal distribution, linearity, and equal error variances.

#### 3.6.3. Predictors of High AI Dependence

A multiple linear regression analysis was executed using sociodemographic markers and trust measures to predict AI reliance (Table 8). The model demonstrated a robust fit, explaining a substantial proportion of the variance (R^2^ = 0.614, Adjusted R^2^ = 0.610, F = 145.32, *p* < 0.0001). Sociodemographic elements, including age group (beta = −0.151, *p* = 0.7529), gender (beta = −0.192, *p* = 0.1630), and education (beta = −0.170, *p* = 0.4642), were not significant predictors of AI reliance. Instead, psychometric trust measures were the primary drivers. With low baseline trust designated as the reference group, a high baseline trust level served as a strong, statistically significant positive predictor of user reliance (beta = 2.149, t = 12.15, *p* < 0.0001).

#### 3.6.4. Sociodemographic Impacts on AI Reliance Scores

To determine whether background context shaped total reliance values, a multiple regression model was developed using only sociodemographic indicators as independent predictors (Table 9). The model included age group, gender, nationality, education level, monthly income, employment status, geographical region, and marital status as predictors. The overall model did not explain a meaningful proportion of the variance in AI reliance scores (R^2^ = 0.036, Adjusted R^2^ = −0.0003, F(23, 603) = 0.991, *p* = 0.475), indicating that sociodemographic characteristics collectively lacked predictive power.

Across all predictors, none reached statistical significance (all *p*-values > 0.05). Age group (*p* = 0.562; F = 0.782), gender (*p* = 0.161; F = 1.968), nationality (*p* = 0.425; F = 0.637), education level (*p* = 0.846; F = 0.167), monthly income (*p* = 0.517; F = 0.814), employment status (*p* = 0.409; F = 0.966), region (*p* = 0.397; F = 1.018), and marital status (*p* = 0.809; F = 0.323) all failed to demonstrate meaningful associations with AI reliance scores.

These findings indicate that AI reliance appears to be a uniform behavioral pattern across demographic groups within this sample, suggesting that sociodemographic background does not meaningfully shape how individuals engage with or depend on AI systems.

## 4. Discussion

The integration of Artificial Intelligence (AI) into the Saudi Arabian healthcare landscape is no longer a small trend but a central component of patient behavior. This study reveals a significant shift where AI, specifically Large Language Models (LLMs), acts as a primary gatekeeper to formal medical consultation. The following discussion synthesizes the findings across behavioral patterns, the “reliance–trust gap”, and the demographic drivers of AI adoption in the Kingdom.

### 4.1. The Paradigm Shift: AI as the New Clinical Front Door

The results indicate a profound shift in the patient’s informational journey. With 92.2% of participants favoring LLMs and chatbots over specialized digital tools, AI has effectively replaced traditional search engines and, for a substantial portion of the cohort, represents the initial step in addressing health inquiries (Table 1). This high frequency of usage (mean = 3.75) aligns with Al-Somali’s findings regarding the role of AI-powered chatbots in advancing health management in Saudi Arabia [8].

The dominance of LLMs over specialized symptom checkers (3%) suggests that the “natural language” interface is a major catalyst for adoption. Patients utilize a conversational interface that mimics human interaction, a trend that underscores the transformative potential of digital health in disease detection and management [2]. Because 71.8% of users report consulting AI as a preliminary step before attending professional appointments, these tools function as an influential “pre-consultation” phase in the modern patient workflow. While this data does not imply that AI replaces institutional healthcare channels or formal clinical triage—metrics that fall outside the comparative scope of this study—it highlights AI’s role as an immediate, self-directed source of health information prior to face-to-face medical encounters.

### 4.2. The “Reliance–Trust Gap”: A Psychological Paradox

A striking finding is the discrepancy between high AI reliance (mean = 15.025) and moderate Treatment Trust (mean = 6.489) (Table 4). Behavioral Habit vs. Clinical Faith: Users have developed a sustained behavioral habit (r = 0.7308), yet they remain cognitively guarded regarding the clinical validity of the output (Table 7). Strategic Hedging: This “reliance–trust gap” indicates that while AI is viewed as a sophisticated assistant, it has not achieved the status of a definitive clinical authority. This reflects a level of skepticism regarding final treatment outcomes without professional oversight, echoing concerns about safety risks when using conversational assistants for medical information [3]. Saudi users demonstrate a pattern of strategic utilization—utilizing AI for rapid screening while reserving final clinical authority for human practitioners [10].

It is important to note, however, that this “reliance–trust gap” was not quantified using a standalone, specialized psychometric instrument. Instead, this construct represents an analytical inference derived from the statistical divergence between two independent, previously validated scales within our framework: the AI reliance subscale [20] and the Treatment Trust Scale [19]. While this conceptual synthesis explains the behavioral tension between high daily utility and guarded clinical faith, future psychometric research should focus on developing unified metrics designed to directly measure this cognitive dissonance.

### 4.3. Economic and Clinical Drivers

The dual motivation of clinical convenience (76.9%) and cost-avoidance (46.7%) creates a distinct adoption profile (Table 2). Efficiency: The high demand for initial treatment suggestions and medication risk comparisons (69.2%) indicates a public emphasis on immediate health literacy. Economic Impact: Nearly half of the respondents utilize AI as a practical cost-avoidance measure, which suggests that AI acts as a technology for patient empowerment, allowing users to navigate health concerns while bypassing professional service fees [12]. However, this economic driver remains secondary to the clinical convenience of rapid information gathering [13].

### 4.4. Demographic Factors

Contrary to many digital divide theories, the analysis shows that age, education, and income do not significantly influence AI-related behaviors in this context (Table 9). The Saudi Context: Adoption in the Kingdom appears uniform across generations, diverging from findings in other regions where younger populations show significantly different perceptions [14]. This uniformity may be attributed to high smartphone penetration and the digital-first infrastructure promoted by the General Authority for Statistics [17]. Gender Significance: Gender emerged as a statistically significant determinant for both AI Reliance (χ^2^ = 11.196, *p* = 0.0037) and AI dependence (χ^2^ = 8.399, *p* = 0.0150), with female participants demonstrating a higher baseline prevalence in the high-frequency categories (Table 6 and Table 9). Following the primary research objectives of this study, this variation is captured as a baseline demographic predictor; exploring granular intersectional subgroups or evaluating multi-layered interactions within this gender variance fell outside the objective scope of the current framework. Bivariate analyses initially indicated that gender was a statistically significant determinant of standalone AI reliance, with female participants showing a higher descriptive prevalence in high-use categories. However, this effect completely disappears in our multiple linear regression model (Table 8). Although the bivariate analysis results (Table 6) showed a significant association between gender and AI reliance, this effect diminished and became non-significant once entered into the multivariable regression model (Table 9). This indicates that the initial gender association was influenced by shared variance with other sociodemographic factors rather than representing an independent effect. This divergence is not attributable to multicollinearity; all Variance Inflation Factors (VIFs) were well below 2.0. Instead, the pattern reflects a mediation process in which psychometric Trust emerges as the primary behavioral determinant. Trust accounts for the variance that gender appeared to explain in the bivariate test. In practical terms, gender differences in AI reliance within this sample are better understood as downstream reflections of differing trust levels rather than as standalone demographic predictors.

### 4.5. Predictors of Behavior: Trust as the Ultimate Catalyst

The regression analysis confirms that trust levels are the only significant predictors of AI engagement (*p* < 0.0001), while demographic variables fail to reach significance (Table 8). The Trust Threshold: High trust levels have a strong positive impact on reliance (β = 2.149), while low trust levels show a strong inhibitory effect (β = −2.338). Clinical Implications: These results imply that psychological factors and the perceived reliability of the system are far more influential than baseline demographics [20]. To bridge the “reliance–trust gap”, stakeholders should focus on improving source attribution and citation in AI-generated advice, which has been shown to bolster user trust [19].

### 4.6. Methodological Scope and Limitations

The cross-sectional nature of this study limits the ability to infer any causal relationships. In addition, the model excluded several important determinants of technology adoption, such as user-experience design, system latency, privacy concerns, perceived risk, prior negative encounters, and the transparency of AI algorithms.

The reliance on online convenience sampling produced a strong demographic skew toward younger individuals and students, which restricts the generalizability of the findings to the wider Saudi population.

The data are also dependent on self-reporting and recall biases. Moreover, the study did not evaluate participants’ digital health or AI literacy, their capacity to detect misinformation, or whether they sought confirmation of AI-generated advice from healthcare professionals.

Because the study lacked direct comparisons with clinic visitation patterns or hospital triage records, the results reflect digital information-seeking behaviors rather than evidence of a systemic shift in how people enter the healthcare system.

Finally, the study did not include any clinical auditing of the AI outputs; the accuracy and medical safety of the recommendations were not assessed. Future work should integrate behavioral data with clinical evaluations to identify potential safety risks.

### 4.7. Recommendations

This study recommends shifting from cross-sectional surveys to longitudinal tracking to definitively establish causal relationships between user trust and behavioral reliance over time.

It also suggests combining self-reported behavioral tracking with clinical auditing of AI outputs to measure the objective medical accuracy of advice and assess user exposure to misinformation.

## 5. Conclusions

Among the parameters evaluated in this study, psychometric trust, rather than sociodemographic background, is the primary driver of AI-driven personalized healthcare engagement among surveyed young adults. While unmeasured behavioral habits and technical system features likely influence adoption, the high levels of reliance on AI for initial diagnosis and treatment planning—coupled with a notable ‘reliance–trust gap’ where users remain skeptical of AI’s professional replacement—suggest that AI is currently functioning as a sophisticated clinical assistant rather than a substitute. These findings imply that healthcare policy and digital health implementations should focus on fostering psychological trust and ensuring the accuracy of AI-driven preliminary screenings, as economic drivers and convenience continue to accelerate the transition toward self-directed digital health management.

## Figures and Tables

**Table 1:** Usage patterns of AI tools for personalized diagnosis and treatment support (*n* = 627).

Item	Response Pattern Category	Frequency (*n*)	Percentage (%)
Frequency of AI tool usage for health-related decision making.	Daily	165	26.3%
	Several times a week	233	37.2%
	Once a week	45	7.2%
	Occasionally (monthly or less)	136	21.7%
	Rarely or never	48	7.6%
Specific categories/types of AI tools utilized for health inquiries.	Chatbots (e.g., ChatGPT, Gemini)	579	92.2%
	Health and fitness applications	143	22.8%
	Symptom checkers	19	3.0%
	Health recommendation systems	29	4.6%
Utilization of AI tools for self-diagnosis purposes.	Yes	449	71.6%
	No	178	28.4%
D-AI tool usage as a preliminary step prior to medical consultations.	Yes	450	71.8%
	No	177	28.2%
Use of AI as a cost-avoidance measure for professional medical services.	Yes	293	46.7%
	NO	334	53.3%

Note: Percentage in section B may exceed 100% due to multiple-choice responses.

**Table 2:** Determinants influencing AI adoption for personalized diagnosis and treatment decisions (*n* = 627).

Reason	Response	Frequency (*n*)	Percentage (%)
Assisting with self-diagnosis	Yes	449	71.6%
	No	178	28.4%
Consultation before visiting a doctor	Yes	450	71.8%
	No	177	28.2%
Avoiding the cost of doctor visits	Yes	293	46.7%
	No	334	53.3%
Comparing medications and understanding risks	Yes	434	69.2%
	No	193	30.8%
Obtaining initial treatment suggestions	Yes	482	76.9%
	No	145	23.1%
Seeking lifestyle and nutrition recommendations	Yes	458	73%
	No	169	27%

**Table 3:** Behavioral engagement patterns in AI-driven personalized diagnosis and healthcare decision making (*n* = 627).

Variable/Statement	Measurement	Result (*n* = 627)
Frequency of AI tool usage for health-related decision making.	Mean Score (1–5)	3.75 (High)
Specific categories of AI tools utilized (e.g., LLMs, Chatbots, symptom checkers).	Most Frequent	(92.2%)
Utilization of AI tools for self-diagnosis purposes.	Agreement (%)	71.6% (Yes)
AI tool usage as a preliminary step prior to medical consultations.	Frequency (%)	71.8% (Yes)
Use of AI as a cost-avoidance measure for professional medical services.	Agreement (%)	46.7% (Yes)

**Table 4:** Distribution of user trust, reliance, and dependence in personalized healthcare AI domains.

Domain	No. of Items	Max. Score	Mean	Std. Deviation	Level
AI Reliance	4	20	15.025	3.24	High
AI Dependence	4	20	14.695	3.63	High
Treatment Trust	4	8	6.489	0.92	Moderate

**Table 5:** User reliance and dependence levels on AI for personalized healthcare decision making.

Level of Engagement	Range of Scores	Reliance Frequency (*n*)	Reliance (%)	Dependence Frequency (*n*)	Dependence (%)
Low	4–9.33	36	5.8%	56	8.9%
Moderate	9.34–14.66	216	34.4%	222	35.4%
High	14.67–20	375	59.8%	349	55.7%
Total	—	627	100%	627	100%

**Table 6:** Sociodemographic determinants of AI reliance levels in personalized healthcare (*n* = 627).

Variable	Variable	High Reliance	Moderate Reliance	Low Reliance	χ^2^	*p*-Value
Gender	Male	126	73	22	11.196	0.0037 *
	Female	249	143	14		
		264	139	22		
	25–34	61	35	9		
	35–44	24	25	3		
	45–54	22	12	2		
	55–64	3	4	0		
	65 and above	1	1	0		
Education	High school or less	137	72	12	2.694	0.6103
	Diploma or Bachelor’s	207	119	19		
	Master’s or PhD	31	25	5		

* *p* < 0.05.

**Table 7:** Pearson correlation matrix for AI reliance, dependence, and Treatment Trust in healthcare decision making.

Variables	AI Reliance	AI Dependence	Treatment Trust
AI Reliance	1		
AI Dependence	0.7308 **	1	
Treatment Trust	0.3843 **	0.4476 **	1

** *p* < 0.01.

**Table 8:** Linear regression analysis: predictors of AI reliance in health management (*n* = 627).

Predictor	B (Estimate)	SE (Std Error)	τ (t Ratio)	*p*-Value	VIF
Constant	13.912	0.467	29.74	<0.0001 *	-
Age Group	−0.151	0.479	−0.31	0.7529	3.16
Gender	−0.192	0.138	−1.40	0.1630	1.06
Education	−0.170	0.233	−0.73	0.4642	1.27
Trust Level (high)	2.149	0.176	12.15	<0.0001 *	1.29
Trust Level (low)	−2.338	0.233	−10.03	<0.0001 *	1.29

Model Statistics: R2 = 0.231, Adjusted R2 = 0.219, F (10, 616) = 18.54, * *p* < 0.0001. Note: VIF values for all predictors were <3.16, indicating no multicollinearity. Residual diagnostics showed a normal distribution and constant variance.

**Table 9:** Multiple regression model: the impact of sociodemographic on AI reliance scores (*n* = 627).

Predictor	DF	Sum of Squares	F Ratio	*p*-Value
Age Group	5	41.196	0.782	0.562
Gender	1	20.734	1.968	0.161
Nationality	1	6.707	0.637	0.425
Education Level	2	3.518	0.167	0.846
Monthly Income	4	34.287	0.814	0.517
Employment Status	3	30.505	0.966	0.409
Region	4	42.891	1.018	0.397
Material Status	3	10.206	0.323	0.809

Model statistics: R^2^ = 0.036, Adjusted R^2^ = −0.0003, F(23, 603) = 0.991, *p* = 0.475. Note: “Low Trust” was used as the reference group. The multiple regression model was adjusted for age group, gender, nationality, education, income, employment, region, and marital status. None of these sociodemographic factors significantly predicted AI reliance scores (all *p*-values > 0.05).

## Data Availability

The raw data supporting the conclusions of this article will be made available by the authors on request.

## Supplementary Materials

The following supporting information can be downloaded at: https://www.mdpi.com/article/10.3390/healthcare14131954/s1. File S1: Survey on the Use of AI-Based Health Tools and Health Decision-Making Among Adults; File S2: Data for Reliance on AI in diagnosis.

## Abbreviations

The following abbreviations are used in this manuscript:

AI	Artificial Intelligence
LLM	Large Language Model
ANOVA	Analysis of Variance
JMP	John’s Macintosh Project (SAS Statistical Software)
SAS	Statistical Analysis System
