# Supplementary material for: Predictors of Trust and Engagement in Personalized Healthcare: A Study of AI-Driven Diagnosis and Treatment in Saudi Arabia
Source: Healthcare (Basel). 2026 Jul 2;14(13):1954. doi: 10.3390/healthcare14131954 (PMC13361430; doi:10.3390/healthcare14131954)
Supplement: Supplementary file 1 [file healthcare-14-01954-s001.zip › healthcare-4392412-supplementary.pdf]

Survey on the Use of AI-Based Health Tools and Health Decision-Making Among Adults

Informed Consent

You are invited to participate in a research study aimed at exploring the prevalence, perspectives, and factors related to health decision-making, with a specific focus on the use of and reliance on Artificial Intelligence (AI) tools among community members in the Kingdom of Saudi Arabia. The study aims to describe the role of AI in providing access to health information and supporting individuals in making decisions related to their health.

Participation is entirely voluntary and involves completing a self-administered electronic questionnaire that takes approximately 2–4 minutes, without any physical intervention, clinical procedure, or random assignment. All information will remain completely anonymous and confidential. Furthermore, participants can skip any question or withdraw at any time without any penalty.

The study involves minimal risk, limited to the potential for a slight amount of discomfort when thinking about personal health decisions; there are no physical risks associated with participation. Although there are no direct benefits, this research will contribute scientifically and socially by addressing a gap in the local literature and providing data that may help enhance equity and fairness in access to and use of digital health services across Saudi Arabia.

Anonymous data will be collected via Google Forms without any identifying information, and access will be restricted strictly to members of the student research team for academic purposes only. All research team members have completed Good Clinical Practice (GCP) training to ensure adherence to ethical standards. All data will be stored securely on password-protected institutional devices. This study adheres to ethical principles for research involving human participants, ensuring absolute confidentiality and privacy.

Limited assistance from AI tools (ChatGPT, OpenAI) was used solely to improve wording and linguistic structure, while the content, analysis, and final revisions were developed independently by the student researchers.

For any inquiries about the study or participants' rights, you can contact the team via email at:

- **hhabusalih@pnu.edu.sa**
- **amaal79119@gmail.com**

Section 1: Screening & Eligibility

Note: If both answers are "Yes", please continue to the next sections.

1. Are you 18 years old or older? *

- [ ] Yes
- [ ] No

2. Do you use Artificial Intelligence tools like (ChatGPT, Gemini, etc.)? *

- [ ] Yes
- [ ] No

3. Do you work or study in the health/medical field? *

- [ ] Yes
- [ ] No

4. Are you currently admitted as an inpatient in a hospital or residing in a healthcare facility? *

- [ ] Yes
- [ ] No

Section 2: Demographic Information

5. What is your age group? *

- [ ] 18–24
- [ ] 25–34
- [ ] 35–44
- [ ] 45–54
- [ ] 55–64
- [ ] 65 and above

6. What is your gender? *

- [ ] Male
- [ ] Female

7. Nationality: *

- [ ] Saudi
- [ ] Non-Saudi

8. What is the highest level of education you have completed? *

- [ ] High school or less
- [ ] Diploma or Bachelor's degree
- [ ] Master's degree, PhD, or higher

9. What is your average monthly household income (in Saudi Riyals)? *

- [ ] Less than 5,000 SAR
- [ ] 5,000 – 9,999 SAR
- [ ] 10,000 – 14,999 SAR
- [ ] 15,000 – 19,999 SAR
- [ ] 20,000 SAR or more

10. Employment Status: *

- [ ] Student
- [ ] Employed / Working
- [ ] Unemployed / Not working
- [ ] Retired

11. Which region of the Kingdom of Saudi Arabia do you currently live in? *

- [ ] Central Region
- [ ] Eastern Region
- [ ] Western Region
- [ ] Northern Region
- [ ] Southern Region

12. Marital Status: *

- [ ] Single

- [ ] Married
- [ ] Divorced
- [ ] Widowed

Section 3: Use of Artificial Intelligence

13. How often do you use AI tools to make health decisions? *

- [ ] Daily
- [ ] Multiple times per week
- [ ] Once a week
- [ ] Monthly
- [ ] Rarely

14. What types of AI tools do you use to make health-related decisions? (Select all that apply)

- [ ] Chatbots (e.g., ChatGPT, Gemini)
- [ ] Health and fitness applications
- [ ] Symptom checkers
- [ ] Health recommendation systems
- [ ] Other

15. I use AI tools to help me with diagnosis. *

- [ ] Yes
- [ ] No

16. I use AI tools before visiting a medical specialist/expert. *

- [ ] Yes
- [ ] No

17. I use AI tools for health questions mainly to avoid the cost of a formal doctor's appointment. *

- [ ] Yes
- [ ] No

Section 4: Reliability & Attitude

18. I believe that advice provided by AI technologies cannot be trusted unless it is backed by references (such as scientific papers or medical organizations). *

- [ ] Strongly agree
- [ ] Agree
- [ ] Neutral
- [ ] Disagree
- [ ] Strongly disagree

19. I prefer that AI provides me with multiple treatment options, even if they are less accurate, rather than giving a single, definitive answer. *

- [ ] Strongly agree
- [ ] Agree
- [ ] Neutral
- [ ] Disagree
- [ ] Strongly disagree

20. I believe I have sufficient skill and ability to recognize incorrect or misleading health information provided by AI. *

- [ ] Strongly agree
- [ ] Agree
- [ ] Neutral
- [ ] Disagree
- [ ] Strongly disagree

21. Using AI to ask health-related questions makes me feel more in control of my health and quality of life. *

- [ ] Strongly agree
- [ ] Agree
- [ ] Neutral

- [ ] Disagree
- [ ] Strongly disagree

Section 5: Reliance & Dependence

22. I rely on AI systems to boost my confidence when making health decisions. *

- [ ] Strongly agree
- [ ] Agree
- [ ] Neutral
- [ ] Disagree
- [ ] Strongly disagree

23. I use AI tools when searching for health information because they make me feel comfortable and reassured. *

- [ ] Strongly agree
- [ ] Agree
- [ ] Neutral
- [ ] Disagree
- [ ] Strongly disagree

24. I use AI tools to seek reassurance regarding my health concerns. *

- [ ] Strongly agree
- [ ] Agree
- [ ] Neutral
- [ ] Disagree
- [ ] Strongly disagree

25. I use AI tools to review and verify my health decisions. *

- [ ] Strongly agree
- [ ] Agree
- [ ] Neutral

- [ ] Disagree
- [ ] Strongly disagree

Section 6: Treatment & Medical Choices

26. I use AI tools to compare different medications and their alternatives to understand potential risks and benefits before making health decisions. *

- [ ] Yes
- [ ] No

27. I use AI tools to obtain initial treatment suggestions. *

- [ ] Yes
- [ ] No

28. I trust the recommendations of AI tools regarding non-pharmacological treatments (e.g., lifestyle, nutrition). *

- [ ] Yes
- [ ] No

29. I prefer to discuss AI tool recommendations with a doctor before making a final decision. *

- [ ] Yes
- [ ] No
